# Understanding Unlicensed Drug Vendor Practices Related to Childhood Malaria in One Rural District of Uganda: An Exploratory Study

**DOI:** 10.1155/2018/6987435

**Published:** 2018-02-12

**Authors:** Eric Liow, Rosemin Kassam, Richard Sekiwunga

**Affiliations:** ^1^School of Population and Public Health, Faculty of Medicine, University of British Columbia, 2206 East Mall, Vancouver, BC, Canada V6T 1Z3; ^2^Child Health and Development Centre, School of Medicine, Makerere University, P.O. Box 7062, Kampala, Uganda

## Abstract

This study investigated unlicensed drug outlets' practices for the management of malaria in the rural district of Butaleja, Uganda. A qualitative design using semistructured interviews was used. Interviews were recorded, translated, transcribed, and analyzed using thematic analysis. A total of 75 vendors, representing 85% of the outlets in the study area, were interviewed. Most of the vendors were associated with a drug shop type of outfit. About three-quarters reported having completed some level of postsecondary education, but just one-tenth of the vendors had qualifications that made them eligible to apply for a license to operate a drug shop. While most outlets stocked at least one type of antimalarial, only about one-quarter stocked an artemisinin-based combination therapy (ACT), one-quarter expressed a preference for ACTs, and less than one-tenth attested to firmly adhering to the national malaria treatment guidelines on dispensing ACTs as the first-line option. In contrast, nine out of 10 vendors stocked quinine and well over a third stocked antimalarials no longer recommended, such as chloroquine and sulphadoxine-pyrimethamine. Given the ongoing gap between the national malaria policy and unlicensed drug outlet practices, this study calls for greater engagement of unlicensed vendors to improve the management of childhood malaria.

## 1. Background

Malaria remains a major burden for children aged five and under in Uganda [1]. Over the past several decades, management of childhood malaria continued to fall short of Uganda's national 2010/2015 targets that 85% of children under five with suspected or confirmed malaria receive the first-line antimalarial within 24 hours of the onset of symptoms [1–4]. While care delivered through Uganda's public health system remains an important national strategy to manage childhood illnesses, the literature suggests visits to private drug outlets to be as common, if not more common, as visits to public facilities [3]. A 2012 nation-wide survey reported that 56.6% of children with fever received treatment from private-for-profit drug outlets compared with 23.8% from public health facilities [5]. As part of a national effort to achieve equitable access to affordable first-line antimalarial medicines (artemisinin combination therapies, ACTs), recent policies have encouraged governmental cooperation with the regulated private sector to increase the availability of affordable ACTs [6, 7]. Accordingly, several initiatives under the Affordable Medicines Facility-malaria (AMFm) umbrella have been implemented to increase access to low-cost ACTs from the formal private sector since 2009 [8, 9].

The formal private-for-profit drug delivery sector in Uganda is regulated and licensed by the National Drug Authority (NDA). Though the requirements for different private drug outlets vary, all outlets must meet licensure requirements if they are to dispense pharmaceuticals legally [10]. By regulation, pharmacies must be supervised by a registered pharmacist. Licensed pharmacies are permitted to dispense all four classes of scheduled medicines: (1) prescription-only Class A drugs (narcotics), (2) prescription-only Class B (Group I) controlled drugs, (3) nonprescription pharmacy-initiated Class B (Group II) controlled drugs, and (4) nonprescription Class C over-the-counter drugs [11]. Licensed drug shops, on the other hand, are only permitted to sell licensed Class C scheduled drugs, which as of 2008 include ACTs [12]. However, such drug shops are not permitted to stock or sell prescription medicines, such as antibiotics, injectable formulations, and other antimalarials like quinine, chloroquine, or artemisinin monotherapies. Unlike pharmacies, licensed drug shops may be supervised by any one of the following professionals: pharmacy technician, registered/enrolled nurse, comprehensive nurse, registered/enrolled midwife, clinical officer (medical, psychiatric, orthopedic, or dental), public health dental assistant, or anesthetic assistant [10, 13]. Under these regulations, licenses may be revoked at any time if unqualified staff members are found to be dispensing medicines, if unqualified staff members are found to have been left in charge of the outlet, or if the private drug outlet is found to be involved in stocking of medicines that are unauthorized, smuggled, or counterfeit [10].

While the literature has reported great success with improving access to ACTs and diagnostic testing from the regulated private sector, these benefits have been largely limited to regions where the regulated sector is abundant [14, 15]. As has been reported for many rural and remote regions of sub-Saharan Africa (SSA) where the regulated private sector is scarce, a large proportion of households in rural Uganda rely on the unlicensed private drug sector [6, 16, 17]. In addition to filling an important vacuum in drug delivery, their preference is driven by convenience (closer to home, faster services and flexible opening hours, and not requiring prescription), affordability (through credit and less expensive medicines), and regular stock-outs of medication at public health facilities [5, 18–20]. Hence, despite their lack of training, these outlets commonly serve as the initial and sometimes the only source of care for many childhood illnesses.

With the unlicensed sector generally excluded from initiatives aimed at improving the management of childhood illnesses in SSA, several studies have voiced concerns over the quality of care in communities relying on the unlicensed sector [16, 20, 21]. With respect to Uganda, national surveys have found very few unlicensed outlets to stock ACTs [7]. In the rural district of Butaleja where the unlicensed sector is far more prevalent than its licensed counterpart, a district-wide survey found that only one-third of children aged five and under received an appropriate antimalarial treatment [22]. Among those who received any antimalarial, the study found that well over one-third of children were treated with antimalarials obtained from the private sector constituting largely unlicensed drug outlets [22]. While much has been documented about the unlicensed drug sector in SSA, for Uganda, much of this literature dates prior to the advent of the AMFm program in 2009. Hence, despite the importance and prevalence of this sector in rural and remote regions of Uganda, little is known about their day-to-day practices. As part of a larger initiative to identify sustainable community-based solutions to improve the management of childhood malaria in the district of Butaleja, the current study aimed to provide a much needed understanding of unlicensed drug outlets and vendor practices in the designated study areas. Specifically, this paper discusses unlicensed drug vendors' basic characteristics, the range of illnesses they treat, the types of antimalarial medicines they stock, prefer, and recommend, where they obtain their medicines from, and how much they charge for antimalarial treatments.

## 2. Materials and Methods

### 2.1. Study Design

An exploratory qualitative study using semistructured interviews was carried out with unlicensed drug vendors in the district of Butaleja to assess their practices related to malaria management. The study was conducted over a six-week period, from August 1 to September 10, 2011, in 44% of the districts' parishes distributed across all 10 subcounties and two town councils. Ethics approval for the project had been previously obtained from the Uganda National Council for Science and Technology (certificate number: HS 906) and the University of British Columbia's Behavioral Research Ethics Board (certificate number: H10-02909). Only those unlicensed drug vendors who provided written consent to participate were recruited and interviewed.

### 2.2. Setting

The study was conducted in the rural eastern district of Butaleja, Uganda. The study setting has been described in more detail elsewhere [23]. Briefly, the district consists of 10 mostly rural subcounties and two town councils which are designated as urban centers. The population in 2010 was estimated at 214,600, with 41,240 households and an annual growth rate of 3.3% [24, 25]. While peak rainfall generally occurs between May and October, unpredictable rainfall is common, making this region hyperendemic to malaria [25]. As a result, the district typically experiences stable and high year-round malaria transmission that contributes to 46% of outpatient visits and 23% of admissions at health facilities [26]. From 2007 to 2009, malaria was ranked as the highest cause of morbidity in the district [24].

As with the rest of Uganda, Butaleja has a public health infrastructure that is administratively stratified into four levels: (1) at the district level, there is one hospital; (2) at the subcounty level, there are Health Centre IIIs; (3) at the parish level, there are Health Centre IIs; and (4) at the village level, there is Health Centre I, constituting a nonphysical structure composed of volunteer members of Village Health Teams [27]. The national policy of Uganda requires first-line antimalarial treatments be made available to the public cost-free at all levels of the public health system. For uncomplicated malaria, this included ACT in adults and in children four months of age and older and quinine for children younger than 4 months or weighing less than 5 kg [2, 28, 29].

### 2.3. Sampling

The sampling frame for this study included 27 of the district's 66 parishes, selected as study sites for the larger district-wide exploratory research [22]. As a first step, a two-stage census was conducted to enumerate all public health facilities and formal (licensed) and unlicensed private-for-profit drug outlets located in the select parishes. First, with the assistance of the local district health team, all formal public and private sector outlets registered with the district health authority records were identified and their existence was confirmed through field visits. Next, with help from the village local council chairpersons, all unlicensed drug outlets were identified. In total, 88 unlicensed drug outlets were enumerated, serving as the sampling frame for recruitment of study participants. All 88 outlets were visited by the research team and one staff from each outlet was invited to participate; all those who consented were recruited and interviewed. A total of 75 participants (one from each outlet) were recruited. Among those excluded (*n* = 13), five had permanently closed down since the census, two had closed down temporarily, and six were not available even after a second recruitment attempt.

### 2.4. Data Collection

Structured interviews were carried out by trained research assistants who had been recruited for the larger study and were fluent in both verbal and written English and the spoken local dialect of Lunyole. The interview topic guide (refer to Supplementary Material (available here)) explored a variety of topics, such as the drug vendors' qualifications, the range of illnesses they treat, the types of antimalarial medicines they stock, prefer, and recommend, where they obtain their medicines from, and how much they charge for antimalarial treatments. All research assistants underwent a week of training consisting of face-to-face discussions about the research objectives, study methods, questions guiding the interviews, and in-the-field training. The interview questions were created in English and translated into Lunyole during the training period. Both versions were pretested prior to their use in the field. Interviews were carried out in English or Lunyole, according to the participants' preference, and usually occurred on site at the participants' places of operation or at their nearby homes in order to avoid disrupting business. Each research assistant conducted, recorded, translated, and transcribed their interviews. Additionally, using a checklist of medicines commonly sold in Butaleja district, all interviews were supplemented with an on-site inventory of all medications stocked by the outlets. Quality assurance of the transcribed data was provided by senior researchers. They supervised the data collection and regularly cross-checked the transcribed data with the recorded interviews to ensure consistency, accuracy, and completeness.

### 2.5. Data Analysis

Vendor transcripts were analyzed using thematic analysis consistent with the Braun and Clarke approach [30]. The initial coding frame was based on topics used to guide the interviews. As most responses ranged from a few words to short sentences, each data item was given equal attention in the coding process. The key words and phrases were first coded using the QRS Nvivo (version 9.0) qualitative data management software and subsequently compiled into subthemes and meaningful thematic clusters. Relative frequency of thematically similar content was then calculated and reported. Demographic information was entered into Microsoft Excel 2010 software and summarized using descriptive statistics. Consistent with any qualitative research, statistical representativeness was not an aim for this study.

### 2.6. Ethics Approval and Consent to Participate

Ethics approval for the study was obtained from the Uganda National Council for Science and Technology (certificate number: HS 906) and the University of British Columbia's Behavioral Research Ethics Board (certificate number: H10-02909). Only those unlicensed drug vendors who provided written consent to participate were recruited and interviewed.

## 3. Results

### 3.1. Census: Enumerating Unlicensed Drug Outlets in the Study Site

Overall, the private-for-profit drug outlets far outnumbered public health facilities (84.3% versus 13.0%).  Table 1 summarizes the variety of private-for-profit drug outlets present at the parishes studied. Among the 108 private drug outlets tallied, the unlicensed private outlets predominated (81.5%), with three-quarters of outfits consisting of unlicensed drug shops. Other unlicensed outfits, such as mobile vendors, general shops, and market stalls, were also identified, but their numbers may be underreported because of their transient nature.

### 3.2. Characteristics of Participating Unlicensed Drug Vendors

Table 2 summarizes characteristics of vendors participating in this study. Well over half of them were female and almost half were both owners and shop attendants. Although an estimated 70.7% reported having had some postsecondary education, only half (*n* = 36) reported holding any kind of health-related qualification, and an even smaller proportion (14%) had qualifications that made them eligible to apply for a license to operate a drug shop (four nurses and one clinical officer).

### 3.3. Range of Illnesses Treated at Participating Outlets

**Figure 1:**
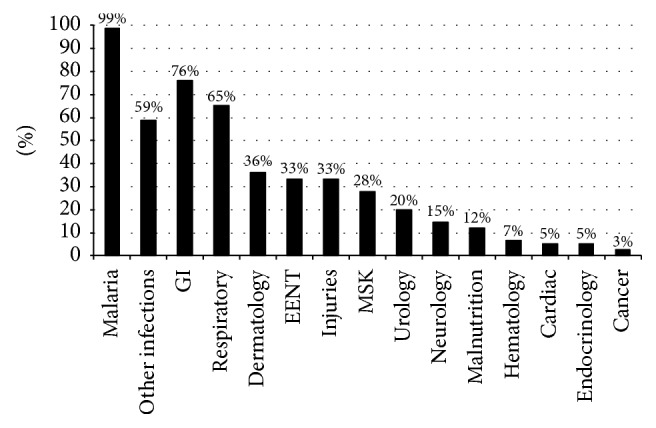
Range of illnesses treated at unlicensed drug outlets (*n* = 75). Drug vendors provided multiple responses. GI: gastrointestinal illnesses; EENT: eyes, ear, nose, and throat illnesses; MSK: musculoskeletal illnesses.

Figure 1 displays the variety of medical conditions vendors said they treated. Malaria was explicitly mentioned by all but one vendor, although the vendor's outlet did stock an antimalarial medicine which the vendor alleged was for treating an unspecified “febrile illness.” Gastrointestinal complaints were the second most common group of disorders treated (reported by 76.0% of vendors), with a large majority citing ailments such as diarrhea (91.2%) and vomiting (75.4%) and a smaller proportion (5.3%) providing vague responses like “stomach problems” and “abdominal pain.” Of those vendors who reported treating respiratory illnesses (65%), 91.8% mentioned treating cough, 75.4% sneezing, 53.1% runny nose/mucus, 38.7% pneumonia, 28.6% asthma, and 14.2% the flu. Among vendors who reported treating other infections (58.7%), 61.4% mentioned syphilis, 47.7% measles, 29.5% typhoid, 27.2% worms, and 20.5% candidiasis and gonorrhea.

### 3.4. Types of Medicines Stocked for the Management of Malaria at Outlets

**Figure 2:**
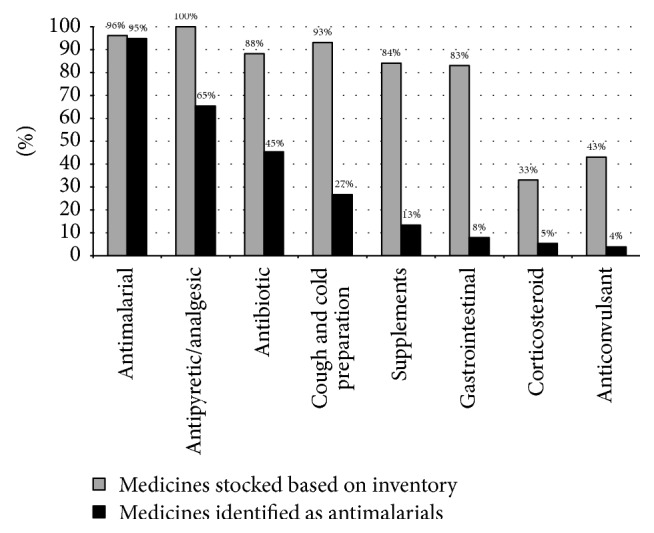
The array of medicines stocked by outlets and those vendors identified as antimalarial (*n* = 75). Drug vendors provided multiple responses. Only those outlets with more than 33% of a stocked medicine are represented in the chart.

Figure 2 shows the variety of medicines stocked by the outlets and displays what vendors identified as antimalarial medicines from their existing stock. Note that only those outlets that carried a minimum of 33% stock of a specified medicine are represented. The on-site inventory revealed that most outlets stocked a wide variety of medicines. The three most common medicines stocked by at least two-fifths of vendors were antimalarials (94.7%), antipyretics/analgesics (65.3%), and antibiotics (45.3%). Paracetamol was among the most stocked antipyretics/analgesics (62.7%). When asked to independently identify antimalarials from their existing stock, several vendors were not able to differentiate between antimalarials and nonantimalarials. While a majority of vendors correctly identified actual antimalarial medicines as antimalarials, almost three-fifths of vendors who stocked an antipyretic/analgesic and half of those who stocked antibiotics incorrectly reported these medicines as antimalarials. Similarly, vendors from the few outlets (4.0%) where no antimalarials were found referred to certain nonantimalarial medicines as antimalarials.

**Figure 3:**
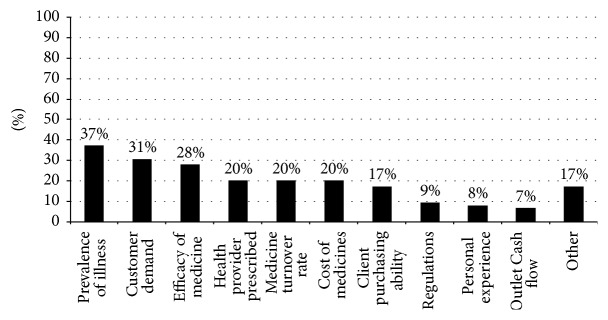
Factors influencing participating vendors' decisions about what types of medicines to stock. Drug vendors provided multiple responses.

Figure 3 summarizes the factors reported as most likely to influence vendors' decisions about which medicines to stock in their outlets. Over a third of the vendors reported the prevalence of an illness in their village to be the most common factor, followed by what the customers requested and what the vendor perceived to be the most efficacious medicine. Medicines prescribed by health providers, medicines that turn over quickly or slowly, and the costs of medicines were reported to equally influence their decisions. A few vendors (17.3%) also mentioned taking into consideration the types of medicines villagers were able to afford; but fewer than 10% mentioned their decisions being influenced by any kind of government regulations.

### 3.5. Contrasting Vendors' Preferences with What They Stocked and What They Sold the Most

**Figure 4:**
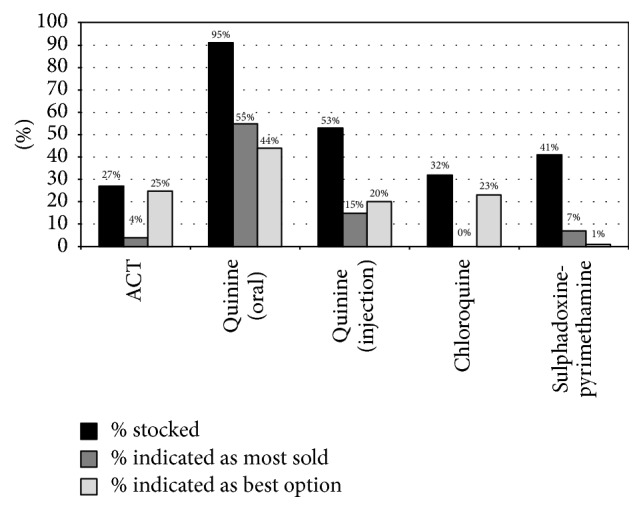
Contrasting vendors' preferred antimalarials with what they stocked and sold the most of (*n* = 75). Drug vendors provided multiple responses.

Figure 4 compares antimalarials that outlets stocked on the day of the interview (based on on-site inventory) with what vendors reported as the most sold antimalarials and what they believed to be the best antimalarial medicine for children aged five and under. While most outlets stocked an antimalarial medicine (Figure 2), only 26.7% stocked an ACT (Figure 4). Across all outlets, only 20% stocked the government recommended ACTs (artemisinin-lumefantrine or artesunate-amodiaquine); the remaining 6.7% of outlets carried other ACTs, such as artesunate-mefloquine, dihydroartemisinin-piperaquine, and artemisinin-naphthoquine. When asked about their preferences, the percentage of vendors who carried an ACT (26.7%) was similar to the percentage that said they considered it to be the best option for treating malaria in young children (25.3%). A substantially lower percentage (4.0%) indicated ACTs as the antimalarial medicines sold most at their outlets.

In contrast, the government's second-line antimalarial quinine (oral tablets and syrup) was stocked by almost all outlets (94.7%). About half the outlets (53.3%) also carried a quinine injection (Figure 4). Just over half (54.7%) of the vendors mentioned oral quinine as the most sold antimalarial medicine and 44.0% stated that they considered it to be the best option for treating uncomplicated malaria in children. The four most common reasons cited for preferring it were that quinine is effective in treating malaria (62.5%), using syrup has benefits (more convenient to stock than suspension formulations and more likely to be taken by children) (18.8%), quinine is safer for children (12.5%), and quinine is easier to stock than ACTs because it is cheaper and more readily available to purchase (9.4%).

The on-site inventory showed that some vendors continued to stock antimalarials that were no longer recommended. For example, chloroquine, which was replaced by artemether-lumefantrine in 2006, was stocked by 32.0% of outlets, and sulphadoxine-pyrimethamine, which is primarily used as chemoprophylaxis for pregnant women and a small subset of children, was stocked for the acute treatment of malaria by 41.3% of outlets [31]. Chloroquine, despite being illegal and ineffective due to high parasite resistance and low cure rates, was considered to be the best option by almost a quarter (22.7%) of vendors. Similarly, artemisinin monotherapies, which are banned, were still being stocked by a small proportion of outlets (6.7%).

### 3.6. What Vendors Routinely Recommended for the Treatment of Malaria in Children Aged Five and under

**Figure 5:**
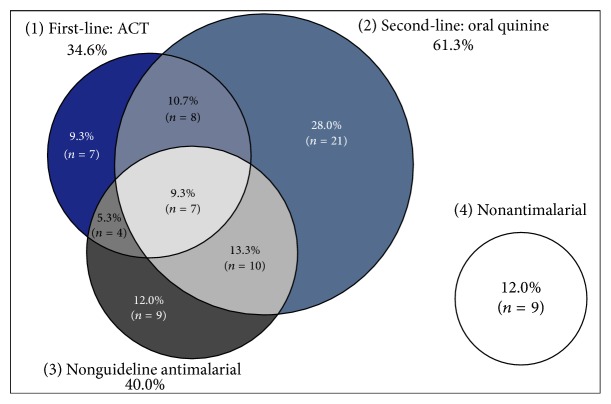
What vendors routinely recommend for the treatment of malaria in children aged five and under. Drug vendors provided multiple responses.

The four circles in Figure 5 represent the four broad categories of medicines vendors reported that they routinely recommended for the management of uncomplicated malaria in children aged five and under. These include (1) first-line antimalarials (ACTs), (2) second-line antimalarials (oral quinine, tablet or syrup), (3) other antimalarials (e.g., chloroquine, sulphadoxine-pyrimethamine, artemisinin monotherapy, or quinine injection), and (4) the use of only nonantimalarial medicines. As shown in Figure 5, oral quinine was the medicine most commonly recommended (61.3%); only seven of the vendors (9.3%) strictly adhered to the national antimalarial policy of recommending an ACT; and about a quarter vacillated between ACTs and the second-line oral quinine or other antimalarials. Additionally (not shown in the Venn diagram), 40.0% of all vendors routinely recommended a variety of nonantimalarials in combination with antimalarials (e.g., combining antibiotics with an ACT). If one were to consider such vendors, then the percentage of those who strictly adhered to the guidelines of using only an ACT falls from 9.3% to 6.7%. In addition, a concerning 52.0% of vendors reported routinely recommending antimalarials that were neither first nor second line for uncomplicated malaria (40.0%), such as quinine injection, artemisinin monotherapy, chloroquine, or sulphadoxine-pyrimethamine, or recommending solely nonantimalarial medicines (12.0%), such as antibiotics and antipyretics. None of the vendors suggested they would treat children under four months of age or lighter than 5 kg differently from those who are older or heavier.

### 3.7. Main Source of Medicines for Drug Outlets

This study found pharmacies to be a common source of antimalarial medicines for these outlets, with about three-quarters (76.0%) of vendors visiting pharmacies in neighboring districts and/or urban centers. Other sources included wholesalers (2.7%), private clinics (2.7%), and other private drug outlets (2.7%). About 16% of vendors who were primarily shop attendants were unsure where the outlet owner obtained the antimalarial medicines from. An estimated 36.0% of vendors reported visiting their choice location three to four times a month, another third (31.9%) visiting only twice a month. Vendors reported that several factors influenced their decisions about where to obtain their antimalarial medicines from. More than half (52.8%) mentioned low prices as the most important factor, followed by convenience (close distance) (33.3%), a reliable inventory (26.4%), incentives offered during purchase of medicines (20.8%), and the quality of the customer service (19.4%).

### 3.8. Prices for Antimalarial Treatments at Participating Outlets

The price of antimalarial treatments at the various outlets varied greatly due to vendors' inconsistent dispensing patterns and the different brands stocked. In addition to selling the full treatment, many outlets frequently sold antimalarial medicines as individual tablets, and several sold antimalarials in combination with other medicines as a part of the full malaria treatment they recommended.  Table 3 summarizes the prices for a full course of antimalarial treatment for a child aged five at participating outlets. In instances where only the price per tablet was shared, this price was used to calculate the price for a full course of treatment based on clinical guidelines [28, 32]. As shown in Table 3, Coartem® was the least expensive of the ACT brands sold, and Falcimon Kit® was the most expensive (although only one outlet stocked Falcimon Kit).

### 3.9. Responsiveness to Clients' Demands

**Figure 6:**
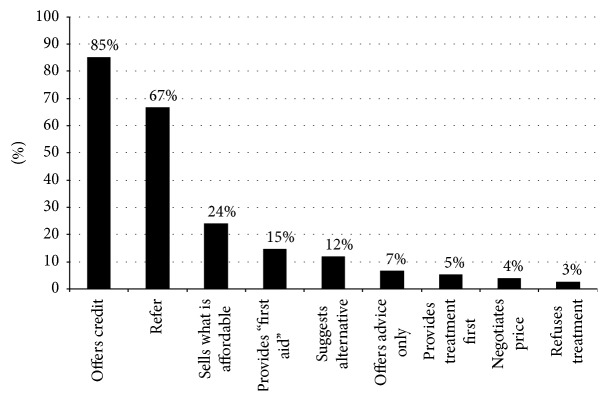
Vendors' actions when clients are unable to afford the recommended antimalarial medicines (*n* = 75). Drug vendors provided multiple responses.

Most vendors reported that they routinely accommodated their clients' requests, with four-fifths of vendors (80.8%) reporting that they would sell exactly what their clients asked for without asking questions. Only 20% of vendors specified that they would ask questions before selling the requested medicines. When vendors were asked what they would do if their clients (typically caregivers of children) could not afford the recommended medicine, as illustrated in Figure 6, the three most common responses were as follows: offer credit (85.3%), refer clients to a public health facility (66.7%), and sell whatever the caregivers could afford (24.0%).

## 4. Discussion

This study describes unlicensed drug vendors' practices related to childhood malaria in rural Uganda. While national outlet surveys have assessed the availability and market share of ACTs within the private-for-profit drug sector, none stratified their data to allow for separate evaluation of the formal and the informal private sector [7, 33]. Additionally, several studies have reported that unlicensed outlets in Uganda provide substandard care, but few have exclusively studied this group's practices since the dissemination of subsidized ACTs at private formal outlets [8]. This study, therefore, provides a unique perspective on unlicensed vendors in one rural district of Uganda. Overall, findings from this study confirm an ongoing gap between the national malaria policy and unlicensed vendors' practices.

This study confirmed that the private sector continues to outnumber public health facilities in rural Uganda [34, 35]. However, unlike many other rural regions, including neighboring districts, the census established that, in Butaleja district, the unlicensed outlets outnumbered licensed private outlets at about 4 to 1, with drug shops being the predominant outfit [34, 35]. Among the vendors who participated, just under three-quarters had some postsecondary education (taken to include vocational training). However, less than half reported receiving any formal health training and only 14% had qualifications that made them eligible to apply for a license to operate a drug shop. Hence, as has been noted in other regions of Uganda and SSA, unlicensed drug outlets in Butaleja are largely operated by staff with little or no medical training [16, 34, 36–38].

Despite operating outside the law, parallel household studies conducted in Butaleja found unlicensed drug outlets to be an important source of antimalarials for children aged five and under [22, 23]. In this study, all vendors attested to treating malaria and/or “fevers of unknown origin” in young children. While an on-site inventory of medicines determined that most vendors stocked some type of an antimalarial, only a quarter stocked an ACT. The limited availability of ACTs at these outlets is surprising, since a national survey of private outlets stocking at least one antimalarial conducted around the same time estimated ACT availability at drug shops (not stratified for licensed and unlicensed status) to be 69.0% and within a subset of unlicensed outlets (that included primarily general retailers) to be 75.6% [33]. Collectively, these findings suggest that while the AMFm initiatives have increased consumer access to ACTs from private licensed outlets in several regions of Uganda, these initiatives have had limited impact on the availability and market share of ACTs at unlicensed outlets in Butaleja.

Findings from this study also raised other important concerns. Most notable for Butaleja is the fact that well over half the vendors interviewed reported using nonantimalarials, such as antipyretics, antibiotics, and cough and cold preparations, alone or in combination, to routinely treat malaria, even when they stocked an antimalarial. Moreover, while a similar proportion of vendors who stocked an ACT also considered ACTs the best option to treat malaria, less than 10% stated that they routinely recommended it, and a mere 4% indicated that ACTs were commonly sold at their outlets. Quinine (oral formulation) was the predominant antimalarial medicine offered by these vendors, despite it being the second-line treatment option. Preference for quinine may stem from the fact that it is cheaper and easier to get and has its use reinforced by that fact that public health providers regularly prescribe it when ACT is out of stock at these facilities [23, 35]. Additionally, about four-fifths of vendors conveyed that they often sold what their clients asked for, suggesting a high desire among many vendors to be responsive to their clients' requests. This is not surprising, as similar to any private business, unlicensed outlets maintain their existence in response to consumer demand [16].

Findings from our study are in agreement with what has been reported elsewhere in SSA, that malaria care provided at most unlicensed drug outlets falls short of the national malaria treatment guidelines [16, 20, 21]. These findings are of immediate relevance to Butaleja, given the unlicensed private drug sector's importance in treating childhood malaria [22, 23]. These studies found only 41% of children to have been treated with an ACT and just 32% treated in accordance with national guidelines, and over half of the caregivers reported problems with obtaining the best antimalarial for their children [22, 23, 39].

While much progress has been made nationally at improving access to free or affordable ACTs, few of these initiatives have trickled down to providers and consumers in Butaleja. For example, the integrated Community Case Management (iCCM) strategy, led by trained community health workers which showed great promise for delivering high-quality care at the community level, has proven to be financially challenging to scale up [40–42]. Consequently, several districts across Uganda, including Butaleja, have yet to benefit from iCCM. Similarly, the national Affordable Medicines Facility-malaria (AMFm) strategy, aimed at increasing the availability of subsidized ACTs from the formal private sector, has been shown to have limited impact in districts, such as Butaleja, where the presence of the formal private sector is negligible [22]. Given the prominence and prevalence of unlicensed drug outlets relative to public and licensed private ones in Butaleja, there is an imperative to improve and align unlicensed drug vendors' practices with national guidelines. Yet, at the policy level, their unrecognized status as health providers makes it a challenge to provide them with the necessary support. It is, however, important to recognize that unlicensed vendors' incentives to thrive may be more than just financial [16]. A parallel qualitative study reported that many such vendors aspire to serve their communities as reputable and honorable citizens, as well as to be viewed as skilled health providers by their peers [43]. In view of this, one should explore the feasibility of bringing such motivated vendors into the formal drug delivery system. This could lessen the gap between practice and policy in rural and remote settings.

Our findings need to be considered in the context of two potential biases. First, as with any study that uses self-reporting, there may have been a tendency for the vendors to respond in a manner that is perceived as socially desirable or likely to avoid litigation by regulatory authorities. However, since the findings actually report a significant deviation from national malaria treatment guidelines, it would appear that such a bias was limited in this study. Second, as with any qualitative study, these findings are intended to illuminate the practice of unlicensed drug vendors operating within the select study parishes. Though the large sample size and inclusion of parishes from across the district suggests that the findings may be illustrative of practice across Butaleja district, further research is needed to confirm whether these findings can be generalized to the whole district. Lastly, over the last several years, there have been a number of initiatives to improve the management of childhood illnesses at formal private-for-profit drug outlets. While it is conceivable that such initiatives may have indirectly influenced the practice of unregulated vendors, there is little evidence in the literature to suggest such diffusion effect. Given that there has been little incentive or opportunity for the unlicensed sector to change their practice, the current findings contribute greatly to this area of research that is scarce.

## 5. Conclusion

The current study provides a baseline understanding of unlicensed drug vendors' practice in a rural eastern district of Uganda. Altogether, findings from this study confirm that both the preference for and the access to subsidized ACTs remain low among such outlets. As unlicensed vendors constitute a large proportion of the healthcare system in Butaleja, they play a critical role in the drug delivery market. This sector of the private-for-profit drug outlets, therefore, has the greatest potential for increasing access to effective antimalarial treatments in rural and remote regions such as Butaleja. Accordingly, engaging and supporting unlicensed vendors to improve their practices may be a practical solution to improve malaria case management and achieve the national targets.

## Figures and Tables

**Table 1:** Distribution of private-for-profit drug outlets at study site parishes across Butaleja (*n* = 108).

Type	Drug outlets	*n* (%)	Total, *n* (%)
Private licensed outlets (formal sector)	Pharmacies	0	20 (18.5)
	Private clinics	2 (1.9)	
	Drug shops	18 (16.7)	

Private unlicensed outlets (informal sector)	Drug shops	83 (76.9)	88 (81.5)
	Mobile vendors	3 (2.8)	
	General shops	1 (0.9)	
	Market stalls	1 (0.9)	

**Table 2:** Characteristics of participating vendors (*n* = 75).

Description	*n* (%)
*Gender*	
Female	49 (65.3)
Male	26 (34.7)
*Unlicensed outlet type*	
Drug shop	70 (93.3)
Mobile vendor	3 (4.0)
Market vendor	1 (1.3)
General shop	1 (1.3)
*Affiliation with drug outlet*	
Owner and shop attendant	42 (56.0)
Shop attendant only	33 (44.0)
*Education*	
Postsecondary (vocational/technical training)^a^	53 (70.7)
Secondary incomplete	6 (8.0)
Primary complete/incomplete, no education	12 (16.0)

^a^36 drug vendors reported having health-related qualifications: nursing assistant/aide (*n* = 27), nurses (*n* = 4), other medical health assistants (*n* = 4), and medical clinical officer (*n* = 1).

**Table 3:** Estimated prices for a full antimalarial treatment at participating outlets.

Medicine	Median price, USh^a^ (range)	Median price, USD^a^ (range)	Response rate^b^
*ACT*			
Coartem	1,500 (1,200–15,000)	0.54 (0.43–5.39)	55.0%
Falcimon Kit	10,000	3.60	
Artenum®	4,000	1.44	
*Nonartemisinin therapies*			
Quinine syrup	3,000 (2,000–4,000)	1.08 (0.72–1.44)	67.6%
Quinine tablets	2,500 (1,000–5,000)	0.90 (0.36–1.80)	
Quinine injection	3,000 (1000–4000)	1.08 (0.36–1.44)	25.0%
Chloroquine	2,500 (1,500–3,000)	0.90 (0.54–1.08)	40.9%
Fansidar®	1,500 (1,500–3,000)	0.54 (0.54–1.08)	25.6%

^a^Price reflects full course treatment. In instances where only the price per tablet was shared, this price was used to calculate the price for a full course of treatment based on clinical guidelines. ^b^The proportion of those vendors who both carried the respective antimalarial medicine and were willing to share what they charged their customers.

## Abbreviations

ACT: Artemisinin-based combination therapy
NDA: National Drug Authority
SSA: Sub-Saharan Africa
AMFm: Affordable Medicines Facility-malaria.

## Supplementary Materials

Supplementary Materials The Supplementary Material contains the set of interview questions used to elicit responses from unlicensed drug vendors discussed in this manuscript. The findings from these responses are presented in the Results section of this publication.
