# Supplementary material for: Understanding Unlicensed Drug Vendor Practices Related to Childhood Malaria in One Rural District of Uganda: An Exploratory Study
Source: J Trop Med. 2018 Feb 12;2018:6987435. doi: 10.1155/2018/6987435 (PMC5830015; doi:10.1155/2018/6987435)
Supplement: Supplementary Materials — The Supplementary Material contains the set of interview questions used to elicit responses from unlicensed drug vendors discussed in this manuscript. The findings from these responses are presented in the Results section of this publication. [file 6987435.f1.docx]

**1. Supplemental Material - Interview Questions**

- What different variety of illnesses do you treat here in your shop?
- Can you tell me and show me the different medicines that you stock. From these, can you identify those that are antimalarial medicines?
- How do you decide which medicines to stock in your shop?
- In your opinion, which is the best option to treat malaria in children five and under? Why?
- Which malaria medicine is sold most in your shop for children five and under? Which is the most common form?
- What malaria medicines do you normally recommend for children five and under? What is the most common medicine?
- Where do you obtain your western medicines from (source)? Why do you go there? How often?
- What is the average cost of the malaria medicine that you recommend?
- Do you always sell to caregivers the antimalarials that they request by name?
- If caregivers cannot afford the malaria medicine you have recommended for their children five and under, what usually happens?
